# Emotional Expression Difficulties, Anxiety, and Their Impact on Digital Communication and Patient Care Among Hospital Nurses

**DOI:** 10.1192/j.eurpsy.2026.10794

**Published:** 2026-07-31

**Authors:** M. G. Bucea, C. Bredicean, M. Stoica, L. Palaghian, G. Covaci, A. Danciu

**Affiliations:** 1 Psychiatry Compartment, Internal Medicine Department, “Dr. Victor Popescu” Emergency Military Clinical Hospital Timisoara; 2 Department of Neuroscience, Psychiatry Discipline, Victor Babes University of Medicine and Pharmacy Timisoara; 3 Psychiatry Compartment, Internal Medicine Department, PhD candidate at Victor Babes University of Medicine and Pharmacy Timisoara, Timisoara, Romania

## Abstract

**Introduction:**

Hospital nurses are frequently exposed to emotionally demanding environments, characterized by high workload and constant professional stress. Difficulties in emotional expression, often associated with partial alexithymia, may affect emotional regulation, anxiety levels, and the quality of communication with both patients and colleagues. In today’s digital era, where social media has become a common channel for emotional interaction, understanding how these affective limitations influence patient care and digital communication is essential.

**Objectives:**

The aim of this study was to investigate the relationship between difficulties in emotional expression, anxiety levels, frequency of online affectionate communication, and need for online emotional validation among hospital nurses. Additionally, we explored the potential impact of these factors on nurse-patient communication and the quality of healthcare delivery.

**Methods:**

We conducted a cross-sectional study on a sample of 125 hospital nurses. Participants completed:

- The GAD-7 scale for anxiety assessment,
- Items from the TAS evaluating difficulty in describing emotions,
- Questions regarding online affectionate communication, time spent on social media, and need for online emotional validation.

Statistical analyses included Pearson correlations and mediation models.

**Results:**

- Difficulties in emotional expression correlated positively and significantly with anxiety (r = 0.42, p < 0.001).
- A significant positive correlation was found between emotional expression difficulties and need for online emotional validation (r = 0.21, p = 0.015).
- No significant associations were identified between emotional expression difficulties and time spent online (r = −0.05, p = 0.64) or frequency of online affectionate communication (r = 0.004, p = 0.96).
- Mediation analyses showed that time spent online does not mediate the relationship between emotional expression difficulties and anxiety.

**Conclusions:**

The findings indicate that difficulties in emotional expression are an important predictor of anxiety among hospital nurses and are associated with a greater need for online emotional validation. These affective limitations may influence the quality of the therapeutic relationship with patients, reducing perceived empathy and emotional support. Although digital communication remains unaffected, it does not compensate for face-to-face interaction difficulties. Implementing training programs focused on emotional awareness and expression could improve both nurses’ mental well-being and patient satisfaction and treatment adherence.

Keywords: anxiety, emotional expression, online validation, hospital nurses, digital communication, patient care

**Disclosure of Interest:**

None Declared

